# The common house spider *Parasteatoda tepidariorum*

**DOI:** 10.1186/s13227-020-00152-z

**Published:** 2020-03-20

**Authors:** Hiroki Oda, Yasuko Akiyama-Oda

**Affiliations:** 1 Laboratory of Evolutionary Cell and Developmental Biology, JT Biohistory Research Hall, 1-1 Murasaki-cho, Takatsuki, Osaka 569-1125 Japan; 2 Department of Biological Sciences, Graduate School of Science, Osaka University, Toyonaka, Osaka Japan; 3 Microbiology and Infection Control, Osaka Medical College, Takatsuki, Osaka Japan

**Keywords:** *Parasteatoda tepidariorum*, Arthropods, Chelicerates, Axis formation, Segmentation, Embryogenesis, Animal evolution, Genome, Database

## Abstract

The common house spider *Parasteatoda tepidariorum,* belonging to the Chelicerata in the phylum Arthropoda, has emerged as an experimental system for studying mechanisms of development from an evolutionary standpoint. In this article, we review the distinct characteristics of *P. tepidariorum*, the major research questions relevant to this organism, and the available key methods and resources. *P. tepidariorum* has a relatively short lifecycle and, once mated, periodically lays eggs. The morphogenetic field of the *P. tepidariorum* embryo is cellular from an early stage and exhibits stepwise symmetry-breaking events and stripe-forming processes that are associated with body axes formation and segmentation, respectively, before reaching the arthropod phylotypic stage. Self-regulatory capabilities of the embryonic field are a prominent feature in *P. tepidariorum*. The mechanisms and logic underlying the evolvability of heritable patterning systems at the phylum level could be one of the major avenues of research investigated using this animal. The sequenced genome reveals whole genome duplication (WGD) within chelicerates, which offers an invertebrate platform for investigating the potential roles of WGD in animal diversification and evolution. The development and evolution of lineage-specific organs, including the book lungs and the union of spinnerets and silk glands, are attractive subjects of study. Studies using *P. tepidariorum* can benefit from the use of parental RNA interference, microinjection applications (including cell labeling and embryonic RNA interference), multicolor fluorescence in situ hybridization, and laser ablation as well as rich genomic and transcriptomic resources. These techniques enable functional gene discoveries and the uncovering of cellular and molecular insights.
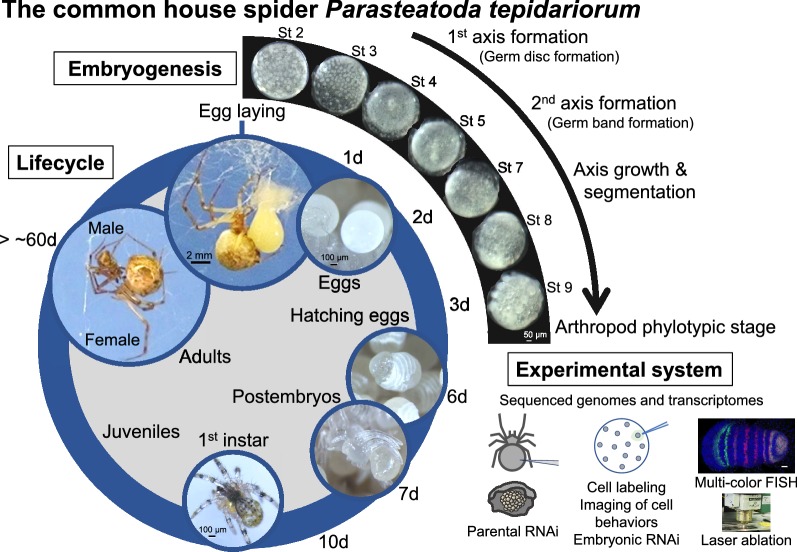

## Natural habitat and lifecycle

**Fig. 1:**
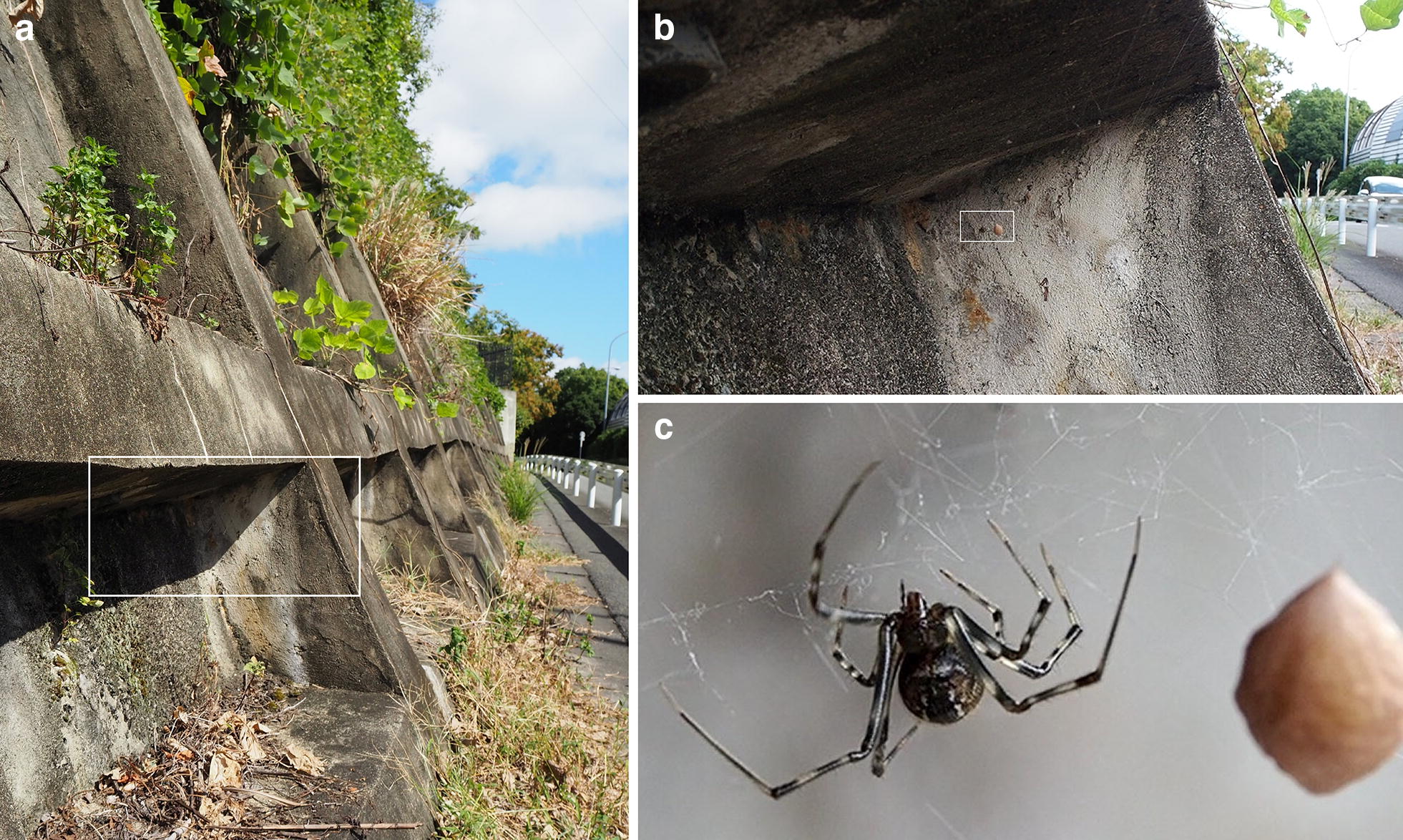
*Parasteatoda tepidariorum* adult female in the wild. **a** Concrete revetment is a typical site for collecting *P. tepidariorum* adults and juveniles. **b** There is an adult female settled on the web in a corner of a concrete revetment (closeup of the boxed region in **a**. **c** Closeup of the wild female with an egg sac (the boxed region in **b**)

**Fig. 2:**
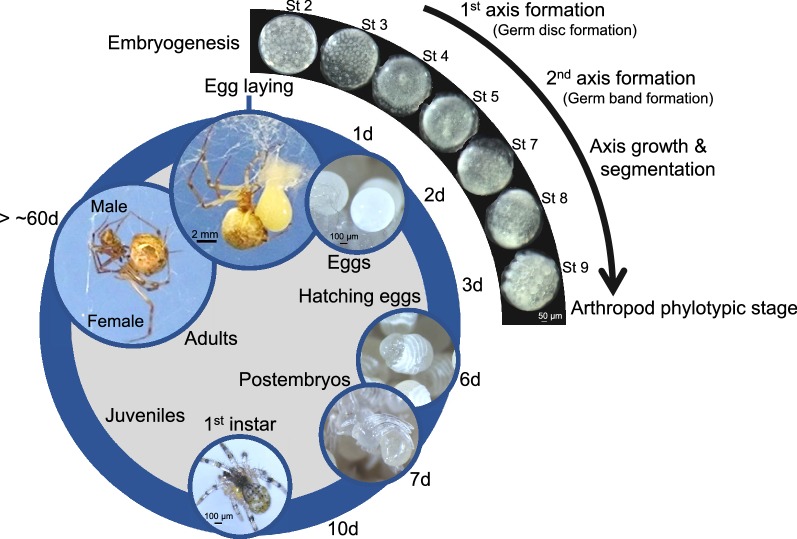
Lifecycle of the common house spider *Parasteatoda tepidariorum*. Except within the inset, all images display animals kept in the laboratory at 25 °C. Days after egg laying (d) are indicated. The images showing embryos are adapted from [8, 17]

*Parasteatoda tepidariorum* (syn. *Achaearanea tepidariorum*), also referred to as the common house spider, has adapted to environments profoundly affected by human activities and is widespread throughout the world. For example, juveniles and adults are found upon their webs in the corners of building surfaces (Fig. 1). Juveniles undergo several molts before becoming adults and displaying sexual dimorphism (Fig. 2). Adult males acquire a slim body type and demonstrate active walking, whereas adult females retain a characteristically large abdomen and stay on the web nest. Once mated, females repeat cycles of egg production and laying (without the need for additional mating). Each cycle takes about 4–6 days in laboratory conditions, and involves the production of an egg sac containing approximately 200–300 eggs. After laying, the eggs hatch in 7 days at 25 °C, and, subsequently, juveniles take approximately 2–3 months to become sexually mature adults (Fig. 2). Of note, *P. tepidariorum* diapauses in short-day conditions [1].

## Lab culture

Laboratory colonies of *P. tepidariorum* can be maintained at 25 °C in long-day conditions (16 h light, 8 h dark) over many (i.e., > 30) generations after capture from the wild. Juveniles and adults are fed twice a week with live *Drosophila melanogaster,* and adult females are additionally fed with live crickets after being transferred to containers for mating and egg laying. Each mated female produces up to ~ 10 egg sacs. When maintaining juveniles hatched from a single egg sac in a 9-cm petri dish with cut cotton, typically only a few will survive due to cannibalism and some of these are then individually transferred into separate *Drosophila* culture tubes where they are reared to adulthood. Videos that illustrate how to culture *P. tepidariorum* in a laboratory setting are available on YouTube (https://www.youtube.com/user/BRHjapan/).

The egg surface is opaque in open air but becomes transparent—without affecting embryonic development—when the eggs are placed in oil (e.g., halocarbon oil 700). This characteristic, combined with the simultaneous oviposition of hundreds of eggs, is helpful for monitoring embryonic development. The development and staging systems for the *P. tepidariorum* embryo have been described [2–5] with some variation in the defined transition points of certain stages. Eggs in oil, however, fail to hatch and no solutions to remedy this issue have been reported.

## Major interests and research questions

### Phylogenetic position

The spider *P. tepidariorum* belongs to Chelicerata, one of the four major superclasses in the phylum Arthropoda. This group has been suggested to be phylogenetically most distant from the Hexapoda (which includes *D. melanogaster*) among extant arthropods [6, 7]. Thus, for understanding the ancestral modes and/or mechanisms of arthropod development, *P. tepidariorum* is a reasonable organism to select for comparative studies.

### Axis formation and segmentation

*Parasteatoda tepidariorum* eggs are spherical in shape. During the initial developmental stages (i.e., stages 1 and 2), no morphological landmarks exist to indicate future body axes. The first symmetry-breaking event leads to the formation of a radially symmetric germ disc, and the second symmetry-breaking event leads to the formation of a bilaterally symmetric germ band (Fig. 2). These symmetry-breaking events are observed as directional collective cell movements or behaviors. Although it is not known whether the direction of the body axes has been determined in advance, a “stochastic” model has been proposed for the emergence of the second axis based on experimental data [8].

The number of segmental units increases while the anterior–posterior axis is growing in the developing *Parasteatoda* embryo. This segmentation mode contrasts with that of *Drosophila*, but is widely observed in arthropods, including many non-*Drosophila* insects. The cellular and molecular events at the posterior terminal region, as well as the presumptive head region of the forming or elongating germ band, in *Parasteatoda* attract researchers who seek to understand the evolution of arthropod segmentation [9–15].

The nature of the morphogenetic field of the early *P. tepidariorum* embryo, in which the embryonic axes and segments are patterned, sharply contrasts with that of the blastoderm embryo of *D. melanogaster*. The *Parasteatoda* embryonic field is cellular at the 16-nucleus stage or perhaps even earlier [16]. In contrast, the field of *Drosophila* consists of a syncytium until the nuclei number increases to approximately 6000. This difference likely correlates with differences in the molecular systems patterning the major embryonic axes [8–10, 17–19].

Periodic stripe patterns associated with the body segments appear to be generated through different mechanisms in the terminal and the middle regions of the *Parasteatoda* embryo. In the terminal regions, unlike *Drosophila*, synchronized oscillation and stripe-splitting occur in concert with axis growth [11–13]. The patterning of the middle region, however, is more *Drosophila*-like, with the simultaneous appearance of segmental stripes. The presence of variability in the insect and spider embryonic fields, in recognition of conserved segmented germ bands (the arthropod phylotypic stage; Fig. 2), fits within the developmental hourglass model [20, 21]. The evolvability of heritable patterning systems associated with arthropod axis specification and segmentation could be the subject of future studies involving *P. tepidariorum*.

### Self-regulation

Another intriguing embryological phenomenon occurring in *P. tepidariorum* is revealed by laser ablation of part of the germ disc, which fragments the field and results in a partial duplication of the body axes [22]. Similar experiments were originally conducted by Holm more than 70 years ago using a different spider species [23]. The results of these experiments suggested the existence of self-regulatory capabilities of fragmented embryonic fields in forming whole-body patterns. There have been few experimental systems, at least in arthropods, used for investigating these types of capabilities in embryonic fields [24–26]. Similar embryological phenomena have been reported in some vertebrate models [27]. *P. tepidariorum* offers an experimental system to study mechanisms of self-regulation.

### Genome evolution

The sequenced *P. tepidariorum* genome reveals a whole genome duplication (WGD) event that possibly occurred in a common ancestor of spiders and scorpions following their phylogenetic separation from the other chelicerate lineages that evolved into mites, ticks, and horseshoe crabs [28]. In addition to duplicated Hox gene clusters, many other developmental genes are found in multiple copies in *Parasteatoda* [29, 30]. This offers a platform for investigating the effects of WGD on the evolution of gene composition, regulation, and interaction, as well as the potential roles of WGD in animal diversification and evolution.

### Lineage-specific traits

The book lungs are a unique respiratory system shared by spiders, scorpions, and their kin. The cheliceral venom gland and the union of spinnerets and silk glands are spider-specific novelties. The development and evolution of these organs, which are associated with the spiders’ terrestrial lifestyles and biology, are intriguing topics to study in *Parasteatoda* [31–34].

### Behaviors

Juvenile and adult spiders exhibit complex behaviors, associated with nest-building, predation, courtship, egg laying, and caring. Despite the importance of this behavioral complexity and diversity from the viewpoint of evolutionary ecology, the development of neural structures and networks underlying such behaviors has been little studied at the cellular and molecular levels. The *P. tepidariorum* experimental system could provide interesting insights in this research area.

## Experimental approaches

### pRNAi

**Fig. 3:**
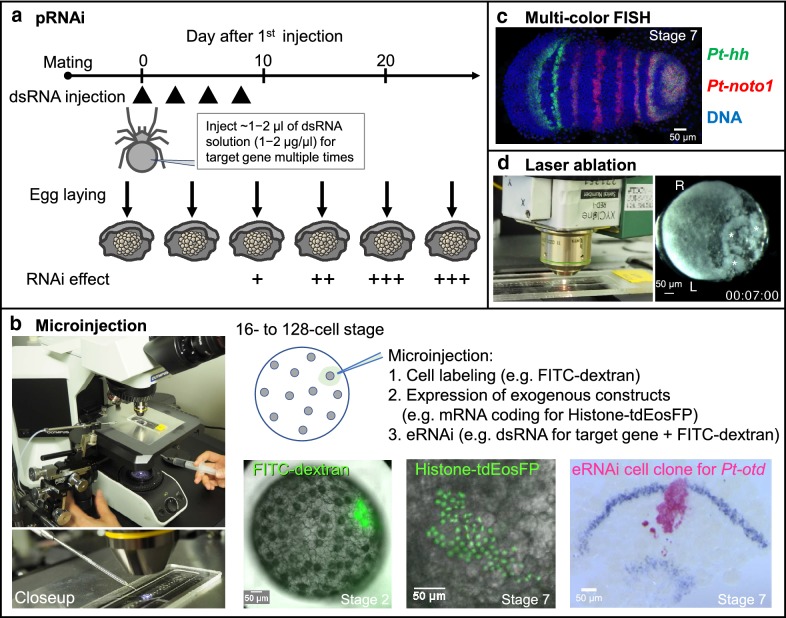
Key methods that have been applied to *P. tepidariorum*. **a** Schematic representation of the typical procedure and timeline of pRNAi-mediated gene knockdown in *P. tepidariorum*. dsRNA for the target gene is repeatedly injected into the abdomen of a mated female. The RNAi effect typically appears in egg sacs that are produced more than 1 week after the first dsRNA injection. **b** The setup for microinjection and three examples of microinjection applications, depending on the materials injected. Bleached eggs are attached on a glass slide using double-sticky tape, covered with halocarbon oil, and then injected. 16- to 128-cell stages constitute the period when the injection can be performed relatively easily. For eRNAi, dsRNA for *Pt*-*orthodenticle* (*Pt*-*otd*) was co-injected with FITC-dextran, visualized in red in the fixed sample. The embryo was also stained for *Pt*-*hh* transcript, demonstrating that *Pt*-*otd* positively regulates *Pt*-*hh* expression in the anterior region of the nascent germ band [11]. The images showing histone-tdEosFP expression and the eRNAi are adapted and modified from Hemmi et al. [13] (CC BY 4.0) and Kanayama et al. [11] (CC BY-NC-SA 3.0), respectively. **c** Flat preparation of a germ band stained for *Pt*-*hh* (green) and *Pt*-*noto1* (red) transcripts by multicolor FISH and counter-stained for DNA (blue). This image is adapted and modified from Hemmi et al. [13] (CC BY 4.0). **d** The infrared laser module used for laser ablation (left) and an example of a laser-irradiated embryo (right). The XYClone laser module is integrated with a 20× objective lens (Hamilton Thorne). In the embryo shown, which had been laser-irradiated 7 h before, dead cells derived from the irradiated region were apparent (asterisks). The left (L) and right (R) embryonic fields were developing into partially separate germ bands. The image of the embryo is adapted and modified from Oda et al. [22] (CC BY 4.0). Scale bars: 50 µm

A key method for genetic manipulation in *Parasteatoda* is parental RNA interference (pRNAi), which is achieved by repeated injections of dsRNA targeting a specific gene into the abdomen of an adult female. Egg sacs produced after 1 week contain hundreds of embryos in which the expression of the target gene is suppressed [17] (Fig. 3a). The specificity of the RNAi effect can be validated by independently obtaining the same phenotypes via the administration of different dsRNAs prepared from non-overlapping regions of the target gene. A variety of phenotypes have been obtained by pRNAi, specific to the target genes, suggesting that functional genetic screens could be implemented to dissect the phenomena of interest. However, the pRNAi approach can be limited if the target genes have critical functions prior to the stages of interest. Hundreds of pRNAi-treated embryos simultaneously developing in an egg sac can be highly useful for conducting downstream experiments, including microarrays and RNA sequencing [11]. Effective combinations of genome-wide gene expression analyses and pRNAi-mediated gene knockdowns may facilitate discoveries of functional genes without relying on knowledge from other organisms.

### Microinjection and eRNAi

Microinjection of single cells in early *P. tepidariorum* embryos has been applied for cell labeling, exogenous protein expression and embryonic RNAi within cell clones (eRNAi) [11, 16] (Fig. 3b). Cell labeling can be used to determine cell fate as well as gene expression dynamics relative to marked cell clones. This can be achieved by microinjection of synthetic mRNA coding for fluorescent marker proteins to track cell divisions and movements by means of live imaging [11, 13, 35]. Freshly laid eggs, within a few hours from laying, are too fragile to be injected, not allowing wider regions of the embryo to be labeled at early stages.

Microinjection of dsRNA in individual cells can also be used to knockdown gene function within cell clones. The gene silencing effect appears to be limited to the injected cell clones, although non-autonomous phenotypic effects might be observed outside of the clones [11]. This clonal eRNAi approach is useful for investigating the roles of genes within small groups of cells, overcoming the deleterious effects of some global pRNAi-mediated gene knockdowns.

### Multicolor FISH

Multicolor fluorescence in situ hybridization (FISH) has enabled the simultaneous visualization and quantification of the expression of up to four different gene transcripts in *P. tepidariorum* embryos [36] (Fig. 3c). This technique could be combined with cell labeling and eRNAi. The use of multicolor FISH can contribute not only to more precise functional analyses, but also to the acquisition of quantitative data for theoretical and mathematical analyses [13].

### Laser ablation

Laser ablation, using a high power, Class 1, 1460 nm infrared laser integrated with a 20× objective lens, has been applied to kill cells in specific regions of the embryonic field without damaging other regions of the embryo [22] (Fig. 3d). This approach may help identify the mechanisms of regulative development in *Parasteatoda* embryos.

### Limitations

To our knowledge, no methods for transgenesis or genome editing have been reported in *P. tepidariorum*, due to difficulties in microinjecting early embryos and limited evidence on the developmental origin of germ cells [37]. Rescue of hatching animals from oil, as well as cannibalism, may be additional hurdles to overcome. Limited availability of antibodies and the lack of spider-derived cell lines are also limiting in this system.

## Research community and resources

There are more than 10 research groups currently using *P. tepidariorum* for evo-devo studies worldwide. They have organized a forum called “SpiderWeb” (http://www.spiderweb.uni-goettingen.de) to share information, opinions and to find opportunities for collaboration.

The genome of a *P. tepidariorum* Göettingen isolate has been sequenced [28] and included in the NCBI’s reference sequence (RefSeq) database (see Table 1). The degree of sequence variation between the genomes of the Göettingen and Osaka/Kyoto isolates is limited [38], thereby allowing most of the *Parasteatoda* community to effectively use the reference genome. There are several sources of gene models and de novo transcriptome assemblies available [28, 38–40] (Table 1). There are several database websites available for *P. tepidariorum* users (Table 1).

**Table 1:** Resources and databases available for *P. tepidariorum* research

Resource/database	Description	Reference/URL/Accession Identifier
GenBank genome assembly Ptep_3.0	Latest version, but with no RefSeq	GCA_000365465.3
GenBank/RefSeq genome assembly Ptep_2.0	16,533 annotated scaffolds	[28] GCF_000365465.2
aug3 gene models	Gene prediction with AUGSTUS	[28] https://i5k.nal.usda.gov/Parasteatoda_tepidariorum
De novo transcriptome assembly	Based on ESTs and NGS reads, mixed sources	[39] http://asgard.rc.fas.harvard.edu/
De novo transcriptome assembly	Based on NGS reads, adult whole body	[40] DDBJ TSA: IAAA01000000
De novo transcriptome assemblies	Based on NGS reads, embryos of mixed stages	[38] DDBJ TSA: IACA01000000, IABY00000000
Developmental transcriptomes	RNA-Seq for successive stages of embryogenesis	[41] GEO: GSE112712
EST clones	5′ ends of 22,812 clones sequenced	[9, 11] FY216297–FY225483; FY368221–FY381845
RefSeq genome assembly (NCBI)	Genome Data Viewer, BLAST	https://www.ncbi.nlm.nih.gov/assembly/GCF_000365465.2/
Common house spider genome project (BCM-HGSC)	Genomic resources	https://www.hgsc.bcm.edu/arthropods/common-house-spider-genome-project
Databases of Genome-based Research (BRH)	JBrowse with EST and RNA-Seq data tracks, BLAST, Protocols, Images/movies	https://www.brh2.jp/
ASGARD (Extavour Lab)	Search the assemblies of arthropod transcriptomes	[42] http://asgard.rc.fas.harvard.edu/

## Data Availability

All data generated or analysed during this study are included in this published article.

## Abbreviations

BCM-HGSC: Baylor College of Medicine Human Genome Sequencing Center
BRH: Biohistory Research Hall
DDBJ: DNA Data Bank of Japan
dsRNA: Double-stranded RNA
eRNAi: Embryonic RNA interference
EST: Expression sequence tag
FISH: Fluorescence in situ hybridization
GEO: Gene expression omnibus
NCBI: National Center for Biotechnology Information
NGS: Next generation sequencing
pRNAi: Parental RNA interference
RefSeq: Reference sequence
RNAi: RNA interference
RNA-Seq: RNA sequencing
URL: Uniform resource locator
WGD: Whole genome duplication
